# Plasmonic Colour Printing by Light Trapping in Two-Metal Nanostructures

**DOI:** 10.3390/nano9070963

**Published:** 2019-07-01

**Authors:** Keith Wilson, Cristian A. Marocico, Esteban Pedrueza-Villalmanzo, Christopher Smith, Calin Hrelescu, A. Louise Bradley

**Affiliations:** School of Physics and CRANN, Trinity College Dublin, Dublin D2, Ireland

**Keywords:** plasmonic colour printing, light trapping, two-metal nanostructures

## Abstract

Structural colour generation by nanoscale plasmonic structures is of major interest for non-bleaching colour printing, anti-counterfeit measures and decoration applications. We explore the physics of a two-metal plasmonic nanostructure consisting of metallic nanodiscs separated from a metallic back-reflector by a uniform thin polymer film and investigate the potential for vibrant structural colour in reflection. We demonstrate that light trapping within the nanostructures is the primary mechanism for colour generation. The use of planar back-reflector and polymer layers allows for less complex fabrication requirements and robust structures, but most significantly allows for the easy incorporation of two different metals for the back-reflector and the nanodiscs. The simplicity of the structure is also suitable for scalability. Combinations of gold, silver, aluminium and copper are considered, with wide colour gamuts observed as a function of the polymer layer thickness. The structural colours are also shown to be insensitive to the viewing angle. Structures of copper nanodiscs with an aluminium back-reflector produce the widest colour gamut.

## 1. Introduction

Unlike traditional color generation methods based on dyes and pigments, the generation of color with nanostructures has become the focus of intense research interest since nanostructures are resistant to photo-bleaching and exhibit superior robustness to chemical, physical and thermal stresses in contrast to dyes and pigments. Moreover, the structural color can be controlled at sub-diffraction-limited dimensions [1], rendering nanostructures as crucial constituents for color generation [2,3,4,5] in a broad range of applications, ranging from color printing [1], color filtering [6,7,8,9], and anti-counterfeit measures [10] to passive reflective display technologies [11] and imaging sensors [12,13]. Thin film stack structures can generate structural color based on constructive or destructive interference, so that only specific spectral regions of the visible light are reflected back to the observer. Other structures based on very thin layers of highly absorbing materials [14] can absorb certain spectral ranges in the visible generating color based on a subtractive color model. However, for these thin film structures achievable colors palettes are often limited. Noble metal nanostructures exhibiting plasmonic modes, i.e., collective electron oscillations at a dielectric-metal interface which can be resonantly excited with visible, near infrared and infrared light [15,16,17], can generate all colors across the visible spectrum through the selective scattering and absorption of light [2,5,18]. Plasmonic modes can be localized or can propagate along the dielectric-metal interface [15,16]. The plasmonic modes are strongly dependent on the noble metal selected, the refractive indices of the surrounding media, the dimensions and shape, as well as on arrangement and distribution, of the nanostructures on a substrate [15,19,20,21]. Structural reflected color generation with structures based on one metallic material has been demonstrated with simple thin film structures [8,14] and periodically nanostructured metallic films [22,23], with all-metal nanostructures [24,25], with nanohole arrays [26], nanoantenna arrays [1,27,28,29,30] or randomly distributed nanoparticles [18,31,32] on top of a back-reflector or on transparent substrates. Plasmonic structural color is attractive for many applications since polarisation dependent [33,34,35,36] or independent [23,27] color generation can be achieved depending on the application requirements, such as stereoscopic printing [35]. Likewise viewing angle sensitive or insensitive [31,37] color generation have been demonstrated. Plasmonic printing technology is becoming more attractive commercially as extreme ultraviolet lithographic processes become more widely available, cheaper and scalable [38,39]. Designs compatible with nanoimprint technology can also offer low cost solutions [40]. Plasmonic color generation based on extraordinary optical transmission [6,41,42], Fano-type resonances [43,44], Wood–Rayleigh anomalies or excitation of propagating surface plasmons *via* grating coupling is highly depended on the viewing angle as well as on the polarisation of the excitation source, which for some application fields, such as displays, is not desirable. Nanohole and pillar-type nanostructures based on gold, aluminium or silver as sole the plasmonic material have been extensively examined to control generated colors and to extend the achievable color palettes [1,28,35]. Additionally, randomly distributed nanoparticles, exhibiting localised surface plasmon modes, have been demonstrated as strong color filters [31]. However, the random distribution of nanoparticles leads to spectrally broad features in the optical response, and consequently to low color clarity. In contrast, patterned nanostructure arrays can exhibit spectrally narrower features due to the uniform shape and spacing of the nanoparticles in an array configuration. Nanostructures exhibiting spectrally narrow modes are of particular interest in structural color generation. Metal-insulator-metal (MIM) nanoparticle array configurations based on a single plasmonic material have been extensively studied for sensing applications in the IR [45,46] and in the visible spectral range [47,48,49] as well as for color generation applications [1,27,29,36,43]. However, the achievable color palette with nanostructures using a sole plasmonic material, for both the nanoparticles and as a back-reflector, is often limited. Extension of the achievable color palette is an important consideration to progress plasmonic color generation as an emerging and viable technology for future devices, such as color filters, displays and anti-counterfeit measures. The color palette might be extended with nanostructures comprising two or more plasmonic materials. Surprisingly, to our knowledge, there have been very few studies on plasmonic color generation with nanostructures comprising two different plasmonic materials [32,50,51,52,53]. Xiong et al. considered random nanoholes in a Cu film separated from an Al layer [50]. Choi et al. reported on a Au coated anodic aluminium oxide template, producing plasmonic nanopores, on top of an Al film [51], while Stewart et al. recently considered two different plasmonic materials, one used for randomly distributed Ag nanocubes and the other as a Au back-reflector [32].

In this work, we report on the design and experimental realisation of hybrid metallic nanostructures for plasmonic color generation by light trapping. Firstly, we consider hybrid structures based on gold nanodisc arrays on top of a back-reflector consisting of a polymer thin film and a 100 nm thick silver film on a silicon substrate. By design, the color generation relies on the interaction of localized plasmonic modes of the nanodiscs with the thin film modes of the back-reflector. In most studies on plasmonic color generation, the color is typically tuned by the simultaneous variation of two fabrication parameters, for example nanoparticle dimension and spacing. Alternatively, color mixing was also achieved by combination of different pixels generating the three primary colors [50]. Here we investigate the simpler possibility to tune the color by varying solely the polymer spacer thickness and show that our hybrid nanostructure can generate an extended color palette. Furthermore, the structural color is viewing angle insensitive, since the major mechanisms for color generation are due to localized modes. Angular dispersive modes play a minor role. Nanoparticle array structures suspended above a continuous metallic layer can act as near perfect absorbers since such structures can exhibit magnetic modes in the visible [54,55,56,57] and IR spectral region [46,58,59,60]. For specific polymer thicknesses (100 nm and 280 nm), our hybrid nanostructures exhibit magnetic modes at around 585 nm, allowing for selective light trapping within the structure, since the magnetic mode cannot couple to the propagating field. Consequently, in the far field, the, otherwise high, reflectance vanishes below 2% in a spectrally narrow window (35 nm). Although thinner films can generate red and purple colors, the spectrally narrow feature at around 585 nm for hybrid nanostructures with certain polymer thicknesses is responsible for the green color generation. Green colors can be only achieved for polymer films thicknesses staring from ca. 80 nm onwards, also typical thicknesses for organic optoelectronic devices. Combinations of gold, silver and copper nanodiscs on gold, silver and aluminium back-reflectors are also considered. Structures of copper nanodiscs with an aluminium back-reflector produce the widest color gamut, occupying 66% of the sRGB triangle. 

## 2. Materials and Methods

### 2.1. Simulations

Simulations were carried out using the commercially available FDTD solver from Lumerical Solutions Inc. (Vancouver, Canada). A broadband plane wave as used for the excitation. The excitation was from the air half-space of the simulation volume, (+z direction in the simulation coordinate system) and p-polarized (parallel to the x-axis). FDTD multi-coefficient fits to experimental data from reference [61] were used for the permittivity of Ag and Si, and to the data from reference [62] for the permittivity of Au. The complex refractive index for the propylene based transparent polymer used in the simulation, was determined by multiple-angle ellipsometry measurements before and after the EBL exposure and chemical lift-off process. The spectral dependences of *n* and *k* are shown in Figure S1a,b, respectively, in the Supplementary Materials. With low absorption, *k*, and a relatively constant *n* value across the visible, it is suitable for thin film interference structures and for maximising the light interaction between the nanodisc array and Ag back-reflector. The boundary conditions in the ±z directions were set to perfect matching layers (PML) optimised for step angles. In the ±x and ±y directions the boundary conditions were set to periodic.

### 2.2. Experimental

Substrates were cut from plane (100) Si wafers (10 cm diameter), to 10 mm × 12 mm sample substrates. The substrates were pre-cleaned by subsequent sonication in solutions of acetone, methanol and isopropyl alcohol (IPA)) (Sigma-Aldrich, Dublin, Ireland) for 20 min each and were dried with nitrogen to ensure no residue remained on the surface. The 100 nm Ag layer was deposited on the substrate using a FC-2000 Electron-Beam Physical Vapour Deposition (EB-PVD) system (Temescal Ferrotec Europe GmbH, Unterensingen, Germany) at a deposition rate of 0.5 Å/s. Immediately after deposition, a thin layer of a propylene based transparent polymer, with commercial name Tafmer PN 2070 (Mitsui Chemicals Group, Tokyo, Japan), was deposited using a simple spin coating process at 3000 rpm for 30 s. 1 wt% and 2 wt% Tafmer in toluene stock solutions were used for the 70 nm and 196 nm thin polymer layer, respectively. The polymer layer acted as a sealant, preventing tarnishing of the Ag. Additionally, it acts as the supporting layer for the Au nanodisc arrays, and was selected due to the resistance of the material to the development process after EBL patterning. In order to pattern the Au discs, a layer of PMMA was spin-coated on top of the Tafmer layer, followed by 2 min annealing at 180 °C on a hotplate. The PMMA was used as the positive tone resist. PMMA layer thickness was measured using a Dektak profilometer (Bruker UK Ltd, Coventry, UK) and was found to have a thickness of 250 ± 5 nm. E-Spacer was spin coated at 3000 rpm for 30 s, and was used to prevent excess charge build up, improving the resolution of finished arrays. The nanodisc arrays were patterned by EBL on a Supra Field Emission Scanning Electron Microscope (FE-SEM, Carl Zeiss, Oberkochen, Germany). An accelerating voltage of 15 kV was used at a working distance of 5 mm, with a beam current of 35.6 pA and aperture of 10 μm delivering an area dose of 250 μC/cm^2^ over an array area of 100 μm. After EBL patterning of the arrays, the E-Spacer was removed by immersion and rinsing in de-ionised water for 10 seconds. The patterns were developed using a 1:3 solution of methyl isobutyl ketone (MIBK) to IPA for 45 s. Samples were immediately submerged in IPA for 10 s to stop the development of the resist, followed by drying with nitrogen. Subsequently, a 100 nm thick Au layer was deposited by EB-PVD, at a deposition rate of 0.5 Å/s. Lift-off was carried out by submersion of the samples for 10 min in Acetone solution heated to approximately 40 °C on a hot plate. This removed the remaining PMMA and unwanted Au from the samples. Before the spectral characterization, the arrays were first examined in reflection with an optical microscope equipped with a 100× objective under bright-field illumination to determine the colour uniformity and eventual surface imperfections. Reflectance spectra were measured using an in-house system. The samples were excited with a xenon lamp under normal incidence using a spot size of approximately 50 μm diameter. Multiple angle scan microspot ellipsometry was carried out on an unpatterned area near the arrays to determine the polymer thickness in the fabricated structures, with a lateral spot size diameter of 300 μm. The fabricated structures were characterized with a Carl Zeiss Ultra FE-SEM Electron microscope (Carl Zeiss, Oberkochen, Germany). An accelerating voltage of 5 kV was used, at a working distance of 4.9 mm and aperture of 10 μm.

## 3. Results and Discussion

**Figure 1:**
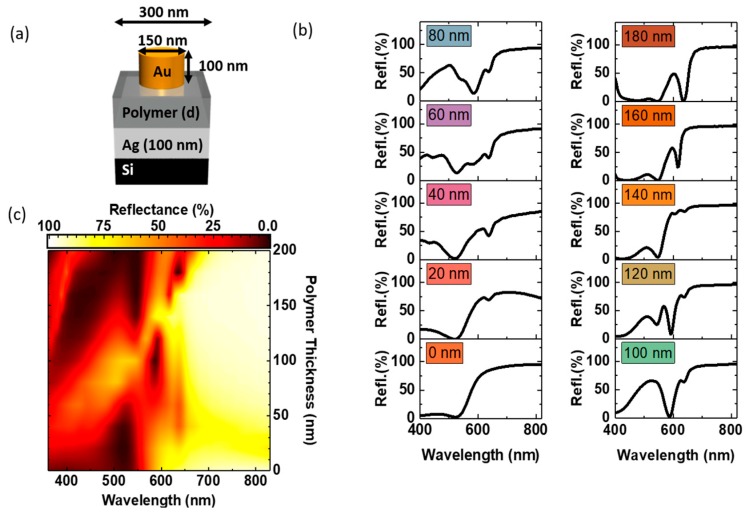
(**a**) Schematic of the hybrid nanostructure unit cell: Square arrays (300 nm pitch) of gold nanodiscs (100 nm high and 150 nm in diameter) on a back-reflector substrate consisting of a polymer thin film on top of a 100 nm thick silver (Ag) layer on silicon. (**b**) Simulated reflectance spectra of the hybrid structures for increasing polymer spacer thicknesses. The individual generated colours (sRGB) are displayed as insets. (**c**) Simulated reflectance as function of polymer thickness.

Firstly, a hybrid metallic nanostructure comprising of Au nanodiscs separated by a uniform dielectric layer from a Ag back-reflector is considered. Figure 1a shows the schematic of the hybrid structure for color generation. The 100 nm high gold nanodiscs with 150 nm diameter are arranged in square arrays with 300 nm pitch on top of a back-reflector consisting of a polymer thin film and a 100 nm thin silver film on a silicon substrate. 

In contrast to pillar-type structures with a small diameter to height ratio, our design is more versatile, easily facilitating the use of different metallic components, and more robust, both during and after fabrication. Moreover, the uniform polymer film protects the back-reflector thin film from degradation. Providing high reflectance, the back-reflector allows for color generation in reflection mode. The dimensions of the gold arrays were chosen according to typical nanofabrication values in order to enable easy fabrication. Besides the easy fabrication aspect, the array period of 300 nm offers two more benefits for color generation. Firstly, since there is a sufficient separation between the nanodiscs, the near field coupling between adjacent nanodiscs is minimal. Secondly, the period is just smaller than the diffraction limit, so our nanostructure does not exhibit diffractive modes and no mode hybridisation [63] is observed for larger periods. Consequently, the spectral response of the hybrid nanostructure should be largely independent of illumination or viewing angle and only weakly dependent on the excitation polarisation, both important considerations for integration in display technologies.

**Figure 2:**
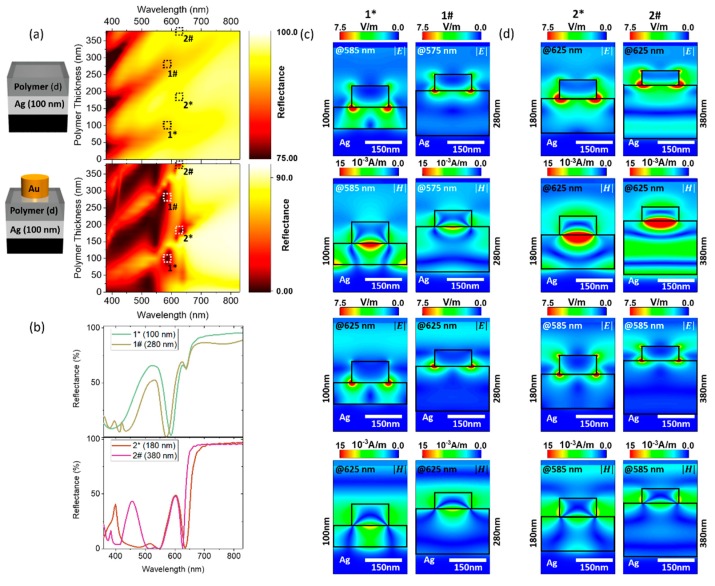
(**a**) Comparison of the reflectance of the back-reflector alone with the reflectance from the Au nanodisc-Ag back-reflector hybrid structure. The occurrence as well as absence of the spectrally narrow features at 585 nm and 625 nm are governed by the interaction of localized plasmonic modes of the nanodiscs with the thin film modes of the back-reflector. (**b**) Simulated reflectance spectra of the hybrid structures for polymer spacer thicknesses of 100 nm (1*), 280 nm (1#), 180 nm (2*) of 380 nm (2#). The spectra are displayed in the individual generated colours (sRGB). (**c**) Electric and magnetic field distributions at 585 nm/575 nm and at 625 nm along the vertical cross section (x–z plane) through one unit cell of hybrid structures with polymer thicknesses of 100 nm and 280 nm. In our coordinate system, the plane wave excitation is normally incident from the airside of the nanostructure, i.e., k vector parallel to the z-axis, and p–polarized, polarization parallel to the x–axis. The black lines in the field maps indicate the gold nanodiscs, the air-polymer and the polymer silver interfaces. (**d**) Electric and magnetic field distributions at 625 nm and 585 nm along the vertical cross section through one unit cell of hybrid structures with polymer thicknesses of 180 nm and 380 nm.

Here we will firstly consider the color palette that can be achieved based on the variation of one parameter, the polymer film thickness. The other structural parameters were fixed at the values shown in Figure 1a. Simulated reflectance spectra of the hybrid structures for polymer spacer thicknesses from 0 to 180 nm, increasing in 20 nm, steps are presented in Figure 1b. In Figure S2 in the Supplementary Materials reflectance spectra for an extended range of polymer spacer thicknesses (0–380 nm) are shown. In order to obtain the color of the nanostructures under daylight illumination, the calculated reflectance spectra were converted to a red, green and blue (RGB) color gamut. Briefly, the spectra are white-balanced against a D65 standard illuminant spectrum [64], and weighted separately against the three equal area integral functions, corresponding to the CIE XYZ 2° standard observer functions [65]. Subsequently the CIE XYZ values are converted [66] and gamma corrected for standard RGB (sRGB) values [67] (c.f. Supplementary Materials for a detailed description). The sRGB colour model is a widely used additive model using three primary colours (red, green and blue) in a set of three 8-bit numbers ranging from 0–255. The sRGB values combine the luminance and the chromaticity of a colour. For example, a sRGB of 255:4:4 indicates a predominantly red colour of maximum luminance, and so would be visible as a bright, pure red colour to the human eye. A value of 63:1:1 also represents a red colour of the same chromaticity (as the relative weighting is the same) but at a quarter of the luminance, and so would be visible as a darker red colour. The generated sRGB colors are displayed as insets. The corresponding sRGB values are given in Table S1 in the Supplementary Materials. For a better visualisation, the simulated reflectance is replotted as a function of polymer thickness in Figure 1c. One remarkable feature at around 585 nm is observed for polymer thicknesses between 80 nm and 120 nm; the otherwise high reflectance vanishes (below 2%) in a spectrally narrow window (35 nm) indicating effective light trapping with in the structure. This spectrally narrow reflectance minimum starts to emerge for polymer thicknesses above 80 nm, reaching it minimal value at 100 nm and disappearing again for polymer thicknesses above 140 nm. Another spectrally narrow feature at around 625 nm can be observed first as small dip in the reflectance spectra for polymer thicknesses between 20 nm and 140 nm and then as pronounced minimum for a polymer thickness of 180 nm. Similar trends can be observed if the polymer thickness is further increased (Figure 2a), i.e., the spectrally narrow feature at around 585 nm (Area denoted as 1* in Figure 2a) can be re-observed for polymers thickness of 280 nm (1#), which again disappears if the polymer thickness is further increased by 40 nm. The spectrally narrow minimal reflectance feature at around 625 nm observed for a polymer of 180 nm (Area denoted as 2* in Figure 2a) can be re-observed also for a polymer thickness of 380 nm (2#). The corresponding reflectance spectra are presented in Figure 2b. The comparison of the reflectance from the hybrid structure with the reflectance of the back-reflector alone (Figure 2a) indicates that the occurrence as well as absence of the spectrally narrow features at around 585 nm (1* and 1#) and 625 nm (2* and 2#) are governed by the interaction of localized plasmonic modes of the nanodiscs with the thin film modes of the back-reflector. In particular, the occurrence of the spectrally narrow minimum at 585 nm, such as observed for the 100 nm polymer film thickness, occurs if the localized Au nanodisc modes spectrally overlap with the local minima observed in the reflectance of the back-reflector (c.f. Supplementary Materials Figure S3). If the localized Au nanodisc modes spectrally overlap with the local maxima observed in the reflectance of the back-reflector, the spectrally narrow feature at 585 nm is absent (c.f. Supplementary Materials Figure S4). The occurrence and absence of the minimal reflectance feature at around 625 nm, as observed for the polymer of 180 nm thickness, shows similar dependencies on the spectral overlap with the local maxima and minima of the reflectance of the back-reflector (c.f. Supplementary Materials Figures S3 and S4).

Before we discuss the origins of the spectral feature at 585 nm in more detail, we want examine further the similarities and differences of the spectrally narrow features observed for different polymer thicknesses. Figure 2c,d show the electric and magnetic field distributions along the vertical cross section (x–z plane) through one unit cell of hybrid structures with polymer thicknesses of 100 nm, 280 nm, 180 nm and 380 nm. Comparing the electric and magnetic field distributions for the hybrid nanostructures with 100 nm and 280 nm polymer thickness in Figure 2c, one notices that the local field distributions are extremely similar, although the polymer thicknesses is increased substantially. The electric fields (at 585 nm/575 nm and at 625 nm) for the nanostructure with 100 nm and 280 nm polymer thickness are stronger at the nanodisc polymer interface than at the nanodisc air interface. The electric fields exhibit multipolar character so that the coupling to the far field is extremely weak. Moreover, the magnetic fields at 585 nm/575 nm are confined within the polymer layers, so that the light can be nearly completely trapped within the hybrid nanostructure. Consequently, the reflectance at 585 nm is minimal.

In contrast, the reflectance is substantially higher at 625 nm, since the electric and magnetic fields couple now to the far field. At this wavelength, the reflectance seems dominated by the back-reflector mode, which at 625 nm exhibits a local maximum. Additionally, the electric and magnetic field amplitudes at 625 nm are smaller than at 585 nm, therefore the light trapping within the nanostructure is weaker. The hybrid nanostructures with 180 nm and 380 nm polymer thickness exhibit also very similar near-field distributions at the nanodisc air and air polymer interfaces (Figure 2d). At 625 nm, the light is completely trapped within the hybrid nanostructure, since the electric fields exhibit multipolar character and the magnetic fields are mostly confined within the polymer layers. However, additional electromagnetic modes can be seen in both; the electric and the magnetic field distributions of the hybrid structure with a polymer thickness of 380 nm. We attribute these modes to the back reflector modes. The near field distributions Figure 2c indicate that the re-occurrence of the spectrally sharp features also depends on the spatial separation of the localized nanosdisc modes with the local maxima and minima of the back reflector modes. The electric and magnetic field distribution at 585 nm reveal another aspect of the interactions between the back-reflector modes and the localized plasmon modes of the Au nanodiscs. As discussed previously, the back reflector modes exhibits a local maximum at around 585 nm for a 180 nm thick polymer film. Due to the interaction between the back reflector modes and the localized modes at 585 nm, the electric field distributions exhibit a dipolar character. Coupling to the far field is now facilitated, which results in a higher reflectance than at 625 nm. 

The spectrally narrow feature at 585 nm is crucial for the generation of green colours, since the spectrally narrow feature overlaps with the maximum of the red CIE XYZ 2° standard observer function z(λ) at 585 nm. The extremely low reflectivity compensates the maximum of z(λ). Moreover, for the generation of bright green colors, a high reflectance between 500 and 550 nm is desirable. At 545 nm, the back reflector modes are the main mechanism responsible for the relative high reflectivity. (c.f. Supplementary Materials for a detailed description of the main mechanisms responsible for the color generation). The spectral narrow feature at 585 nm does not occur, if the polymer thickness is below 80 nm or the Ag film is not present in the hybrid structure (c.f. Supplementary Materials). Green colours can be only generated with hybrid nanostructures with certain polymer thicknesses (above 80 nm), since the spectral narrow feature arises from both, the spectral and spatial overlap of the localized plasmonic modes of the nanodiscs and the local minima of the back reflector modes.

**Figure 3:**
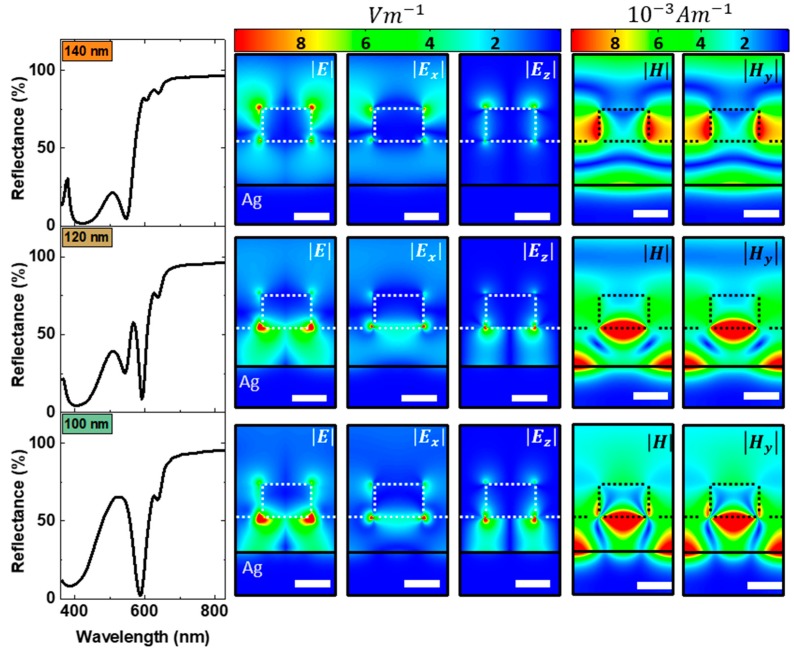
Electric and magnetic field distributions at 585 nm along the vertical cross section (x–z plane) through one unit cell of hybrid structures with polymer thicknesses of 140 nm, 120 nm and 100 nm. In our coordinate system, the plane wave excitation is normally incident from the air side of the nanostructure, i.e., k vector parallel to the z–axis, and p–polarized, polarization parallel to the x-axis. The black line in the field maps indicates the polymer silver interface. The white scale bar is 100 nm.

In order to get more insight into the origin of spectrally narrow feature at 585 nm, we now examine the electric and magnetic field distributions along the vertical cross section (x–z plane) through one unit cell of hybrid structures with polymer thicknesses of 140 nm, 120 nm and 100 nm (Figure 3). The plane wave excitation is polarized along the x-axis and the k vector is parallel to the z direction, i.e., normal incidence. The white scale bars are 100 nm. The black line represents the polymer silver interface. For a polymer thickness of 140 nm, the hybrid nanostructures do not exhibit a spectrally narrow near–zero reflectance dip at 585 nm (Figure 3 left column, upper row). The electric field distributions for the nanostructure with 140 nm polymer thickness exhibit dipolar character, so that a coupling to the far-field is facilitated. Additionally, the total electric field as well as the E_x_ and E_z_ field components are stronger at the nanodisc air interface than at the nanodisc polymer interface. The magnetic field distribution reveals that although there is a strong field localisation between the nanodiscs, the magnetic field couples to the far-field as well. Consequently, the nanostructure exhibits a high reflectance at 585 nm in contrast to the nanostructures with 120 and 100 nm polymer thickness, respectively. Decreasing the polymer thickness to 120 nm leads to light trapping within the hybrid structure. Both, the electric and magnetic fields are now confined within the polymer layer. The field distributions in Figure 3 middle row show very strong localization of light under the nanodiscs. The electric field distribution does not show a dipolar character anymore. We attribute this together with the strong confinement of the magnetic field within the polymer as the major mechanisms responsible for the spectrally narrow near-zero reflectance dip at 585 nm which becomes more pronounced when the polymer thickness is further reduced to 100 nm. In this case, the electric and magnetic fields are even more confined within the polymer layer, indicating efficient light trapping. 

**Figure 4:**
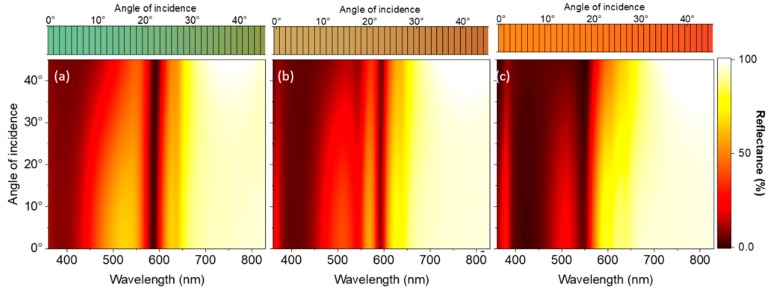
Simulated reflectance spectra as function of the illumination angle for hybrid structures with polymer thicknesses of (**a**) 100 nm, (**b**) 120 nm and (**c**) 140 nm. The excitation was p-polarized. The calculated generated colours (sRGB) corresponding to the individual illumination angles are displayed above the simulated reflectance.

Since viewing angle insensitivity is highly desirable for structural color generation, we studied the angle-resolved optical response of our hybrid structure. This allows us to investigate in detail if the major mechanisms for structural color generation are due to localized modes or due to angular dispersive modes, such as propagating or diffractive modes. Viewing angle insensitivity implies that the color generation is mainly due to localized modes and that propagating and diffractive modes play only marginal roles. Figure 4 shows the calculated reflectance spectra and the generated colors as function of the angle of incidence for p-polarized excitation. Figure 4a shows the angular resolved reflectance of the hybrid structures with 100 nm polymer spacer thickness. Although such spectrally sharp features were previously observed in transmission due to hybridization of grating modes to propagating modes [68,69], here it is clear that the spectrally narrow mode at 585 nm is a localized mode and does not exhibit any angular dispersion. An angle dependent mode is observed in the blue spectral region (400–500 nm). We attribute this mode to the so-called guided mode [63]. The generated colors are barely influenced by the angle of incidence of the excitation, and hence the generated colors are not sensitive to the viewing angle. Figure S9 in the Supplementary Materials shows the reflectance spectra for different angles of incidence as well as optical band structure of a hybrid structure with 100 nm polymer thickness, i.e., the calculated reflectance as a function of the in–plane wavevector and energy for p-polarized excitation.

Increasing the polymer thickness to 120 nm (Figure 4b) leads to the near disappearance of the dispersive guided mode. The localized mode becomes spectrally sharper. For 140 nm polymer thickness the localized mode at 585 nm, and the guided mode observed in case of the 100 nm polymer thickness, do not exist. Instead, a spectrally blue shifted localized mode is observed at 550 nm. The color generation is mainly insensitive to the viewing angle.

**Figure 5:**
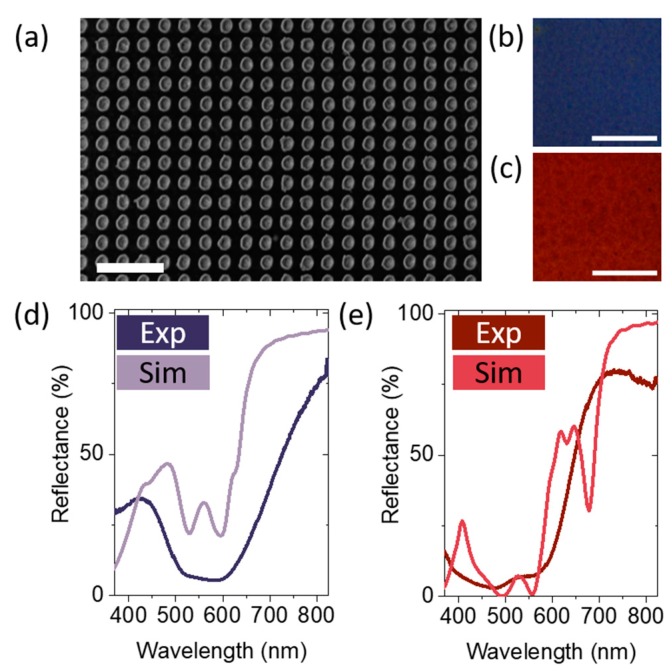
Experimental realisation: (**a**) SEM image of a representative nanodisc array (with 175 nm diameter discs and 300 nm pitch). Scale bar is 1 µm. (**b**,**c**) Optical microscope images (at 100× magnification) for the samples with polymer thicknesses of 70 nm and 196 nm, respectively. The white scale bars are 20 µm (**d**,**e**) Experimental and calculated reflectance spectra for polymer thicknesses of 70 nm and 196 nm, respectively. The spectra are presented in the corresponding sRGB colour.

We turn now our attention to the experimental realization of hybrid nanostructures to demonstrate the feasibility of facile fabrication of the versatile and robust two-metal component hybrid structures. In brief, a 100 nm thick silver film was deposited by evaporation on a silicon substrate. Subsequently, thin films of a propylene based polymer were spin coated on the silver film to form the back-reflector. 1 wt% and 2 wt% Tafmer in toluene stock solutions were used for the 70 nm and 196 nm thin polymer layer, respectively. In order to pattern the nanodisc arrays by electron beam lithography (EBL), a layer of ~250 nm positive tone resist (PMMA) was spin coated onto the polymer layer. After e-beam exposure, the array patterns were developed and a 100 nm thin gold layer was deposited by evaporation. Residual PMMA and gold was removed from the sample via lift-off leaving a 100 µm × 100 µm square array of nanodiscs with 175 nm diameter and 300 nm pitch. A SEM image of a representative array is shown in Figure 5a. Full fabrication details can be found in the Methods section. As a proof of concept, samples with two different polymer thicknesses, (70 ± 5) nm and (196 ± 5) nm, were fabricated. The thicknesses were measured using multiple angle scan microspot ellipsometry on an unpatterned area near the arrays. Distinct blue and red colors are observed for polymer thicknesses of 70 nm and 196 nm, respectively. The corresponding optical microscope images at 100× magnification for the samples are shown in Figure 5b,c. The uniform color distribution in the microscope images indicates a large area structural uniformity across both arrays, in agreement with the SEM images.

The experimental reflectance spectra, presented in Figure 5d,e, were measured using a xenon lamp under normal incidence with a spot size of approximately 50 μm in diameter. The spectra are presented in the corresponding sRGB color calculated from the experimental spectra, in excellent agreement with the observed color in the optical microscope images. For comparison, the calculated spectra for polymer thicknesses of 70 nm and 196 nm are shown in Figure 5d,e, respectively. The simulated spectra are presented in the corresponding sRGB color. Overall, the simulations and experimental findings are in good agreement. The calculated colors are close to the experimental observations. However, the experimental spectra exhibit spectrally broader and less well defined features. This can be attributed mainly to variation in the thickness and surface roughness of the polymer layer, as well as to shape and size variation of the fabricated nanodiscs across the arrays. As discussed previously, the interaction of localized plasmonic modes of the nanodiscs with the thin film modes determines the occurrence of spectrally narrow features, i.e., the spectral and spatial overlap with the minima in reflectance of the back-reflector. The spectrally narrow feature observed at around 680 nm in Figure 5e is strongly sensitive to changes in the spectral positions of the localized nanosdisc resonances, i.e., the red shifts of the resonances, as the disc diameter is increased (c.f. section 8 in the Supplementary Materials). Additionally, the area underneath the discs could have been overexposed during the e-Beam lithography, due to the close spacing of the nanoparticles. As a consequence, the nanodiscs could be embedded in the polymer. A possible optimization of the fabrication procedure involves minimizing the mentioned variations in the thickness of the polymer layer as well as variations in shape and in size of the fabricated nanodiscs across the arrays, i.e., using different polymers or polymer combinations for the polymer thin films.

**Figure 6:**
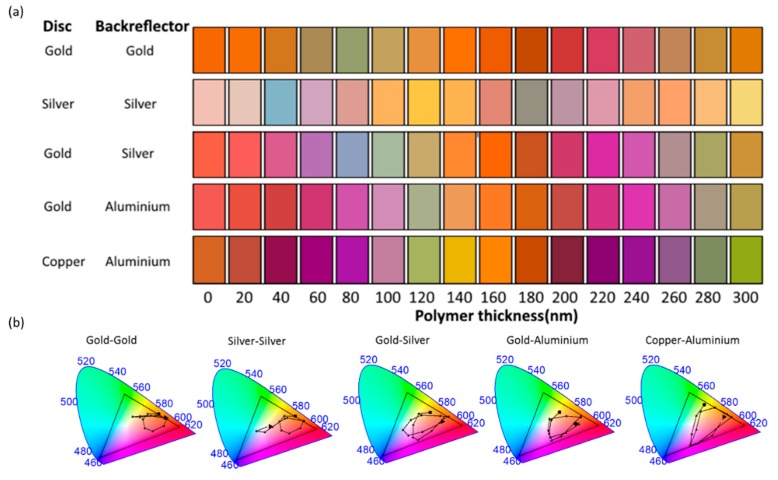
(**a**) Generated colour as a function of polymer layer thickness for different metal disc and back-reflector combinations. (**b**) CIE colour-space maps for each combination as a function of polymer thickness.

Having demonstrated good agreement between the simulated and experimentally generated color for hybrid structures comprised of Au nanodiscs with a Ag back-reflector we now consider different metal combinations. The generated colors from the FDTD simulated reflectance spectra for a variety of structures using combinations of silver, gold, aluminium and copper are shown in Figure 6. Au is a common material for nanostructures due to its resistance to tarnishing as well as its plasmon resonances in the visible range. Nevertheless, the use of Au as a back-reflector and nanodisc material has limitations due to the d band absorption of gold below 520 nm, resulting in low reflectance at shorter wavelengths in the visible spectrum. Consequently, predominantly orange and red tones are generated. The CIE plot shows a concentration of chromaticity values in close proximity to the red trimulus value, occupying 13.56% of the sRGB triangle in the CIE chromaticity diagram. The red, green and blue primaries (corners of the sRGB triangle) represent the positions on the CIE colour-map that have the highest colour clarity. In order for a generated colour to be positioned in close proximity to one of these positions, the nanostructures must exhibit a substantial reflectance intensity in only one of the three colour cone regions of the visible spectrum, while having significantly smaller reflectance in the other two. The comparison of the occupied area on the CIE plot is a measure of distinction between colours in the colour palette, i.e., smaller area, means the colours are clustered, for example in the center of the CIE plot and hence, the color seem all a bit washed out.

For a Ag-Ag system, the generated color palette consists entirely of pastel colors. The generated colors occupy 11.29% of the sRGB triangle in the CIE chromaticity diagram. Furthermore, this combination is less robust, since the Ag nanodiscs could easily tarnish, though the silver back-reflector is protected from the environment by the polymer layer. The Au-Ag hybrid metallic structure, discussed in detail above, produces a color palette which occupies 44.41% of the sRGB triangle in the CIE chromaticity diagram. To explore a cheaper alternative to the Au-Ag hybrid system, we propose to combine materials of lower cost. Aluminium exhibits high reflectance, comparable to Ag. The formation of a self-terminating 2 nm oxide layer protects the material from tarnishing [70], facilitating the fabrication. Replacing the Ag back-reflector with aluminum, produces a color palette with very similar properties to the Au-Ag hybrid metallic structure. However, the achievable color palette is slightly reduced, occupying 38.56% of the sRGB triangle. For the nanodisc material, Cu is an alternative to Au, since Cu nanodiscs exhibit resonances in the visible, at around 600 nm. The reflectance of Cu-Al hybrid structures show similarities to the reflectance of the Au-Ag structures, i.e., well-defined spectral features and high reflected intensity, corresponding to sRGB values with high color clarity (see Figure S15 in the Supplementary Materials). However, the observed modes in reflection are less spectrally sharp, since the plasmon resonances of both aluminium and copper are more damped compared to the plasmons of the more expensive Ag and Au materials. While the Al back–reflector–Cu nanodisc combination generates the largest color range, occupying 65.90% of the sRGB triangle, it must be kept in mind that copper oxidizes in ambient condition and encapsulation/protection of the copper nanodiscs would be necessary.

## 4. Conclusions

We have presented a hybrid metallic nanodisc back–reflector structure capable of producing vibrant color in reflection under white light illumination. The nanodiscs are separated from the continuous film back-reflector by a uniform polymer layer. This type of structure allows for the easy use of different metals for the nanodiscs and back–reflector. In the first instance Au nanodiscs separated from a Ag back-reflector were considered. FDTD simulations showed a large color gamut could be achieved as a function of the polymer film thickness with viewing angle insensitivity. Characterization of fabricated samples showed good agreement of the experimental reflectance spectra with the simulation data. A range of material combinations was investigated. The color palette could be extended with nanostructures comprising two plasmonic materials. The colors generated by structures combining two different plasmonic materials occupy in-between ∼40% and ∼66% of the sRGB triangle in the CIE chromaticity diagram, with the Al back-reflector-Cu nanodisc combination showing the largest color range, while the colors generated by nanostructures consisting of a sole plasmonic material cover less the 14% of the sRGB triangle. 

## Supplementary Materials

The following are available online at https://www.mdpi.com/2079-4991/9/7/963/s1, Figure S1: Complex refractive index of the propylene-based polymer. Table S1: Calculated sRGB values of the generated colors for different polymer thicknesses. Figure S2: Calculated colour generation (sRGB) as a function of polymer thickness. Figure S3: Comparison of the reflectance of the back-reflector alone with the reflectance from the Au nanodisc-Ag back-reflector hybrid structure and the simulated reflectance spectra of the individual components for a polymer thickness of 100 nm. Figure S4: Comparison of the reflectance of the back-reflector alone with the reflectance from the Au nanodisc-Ag back-reflector hybrid structure and the simulated reflectance spectra of the individual components for a polymer thickness of 180 nm. Figure S5: Main mechanisms for the generation of red and orange colours. Figure S6: Main mechanisms for the generation of green colours. Figure S7: Main mechanisms for the generation of blue and violet colours. Figure S8: Left: The calculated reflectance spectra of the back reflector alone of the hybrid nanostructure and of a hybrid nanostructure with the Ag thin film as a back reflector. The generated colour are displayed as inset. Right: The electric and magnetic field distributions at 450 nm, 545 nm, 585 nm and 625nm. Figure S9: Reflectance spectra for different angles of incidence and optical band structure of the Au nanodisc-Ag back-reflector hybrid structure with 100 nm polymer thickness. Figure S10: Calculated colour generation (sRGB) for different nanodisc heights. Figure S11: Calculated reflectance spectra of nanodisc arrays for discs with 125 nm, 150 nm and 150 nm diameter. Figure S12: Comparison of the experimental (stars) and calculated colours (squares) in CIE Plot. Figure S13: Calculated reflectance spectra of nanodisc arrays for discs with diameters of 175 nm and 100 nm in height. The nanodiscs could be embedded in the polymer to some extent, due to overexposure. Figure S14 (a) Polymer thickness dependence and (b) Au nanodisc diameter dependence. Figure S15: Calculated colour generation (sRGB) by a Cu nanodisc-Al back-reflector hybrid nanostructure as a function of polymer thickness.
