# Supplementary material for: Plasmonic Colour Printing by Light Trapping in Two-Metal Nanostructures
Source: Nanomaterials (Basel). 2019 Jul 1;9(7):963. doi: 10.3390/nano9070963 (PMC6669635; doi:10.3390/nano9070963)
Supplement: Supplementary file 1 [file nanomaterials-09-00963-s001.pdf]

Supporting Information for Plasmonic colour printing by light trapping in two-metal nanostructures

Keith Wilson, Cristian A. Marocico, Esteban Pedrueza-Villalmanzo, Christopher Smith, Calin Hrelescu and A. Louise Bradley*

School of Physics and CRANN, Trinity College Dublin, Dublin, D2, Ireland; Keith Wilson
* Correspondence: BRADLEL@tcd.ie;

1. Complex refractive index of the propylene-based polymer

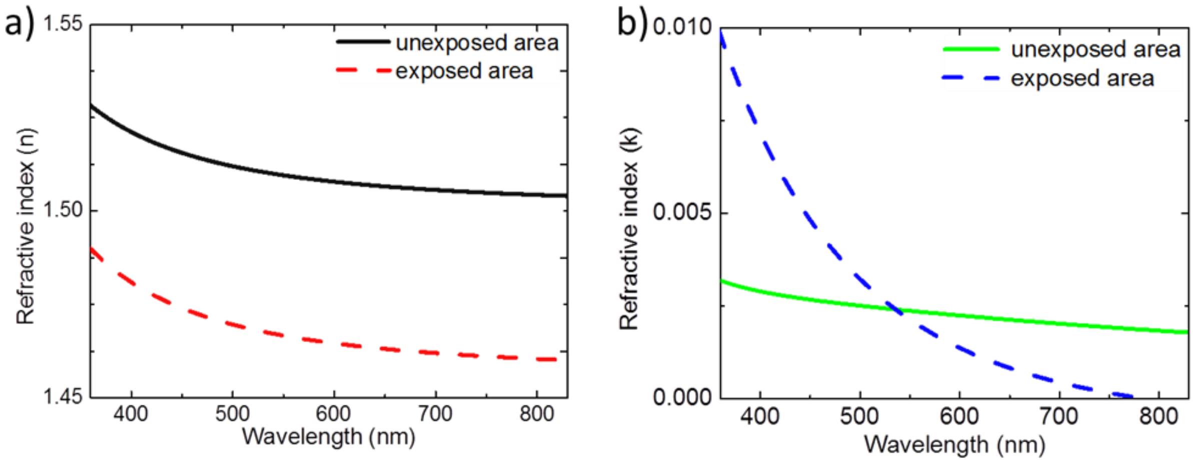

Figure S1: Complex refractive index of the propylene-based polymer (Tafmer PN 2070 – Mitsui Chemicals Group Ltd.) used in nanostructure fabrication measured by ellipsometry for both the EBL exposed area and the unexposed area. Both areas were treated chemically according to the fabrication procedure.

2. Reflectance spectra conversion to sRGB colours

The colour generated by the hybrid nanostructures under daylight illumination was retrieved from the calculated reflectance spectra. The reflectance spectra were converted to the red, green and blue (RGB) colour gamut considering an additive model using tristimulus values of three primary colours (red, green and blue) in a set of three 8-bit numbers ranging from 0-255. In the conversion process the reflectance spectrum, $R(\lambda)$ is white-balanced against a D65 illuminant spectrum $S(\lambda)$ and weighted separately against the three equal area integral functions $x(\lambda)$, $y(\lambda)$ and $z(\lambda)$, corresponding to the CIE XYZ 2° standard observer functions[1]. The $y(\lambda)$ curve describes the luminosity of a colour, fully analogous to the luminous efficiency describing the average sensitivity over all three colour cones to the perception of brightness[2]. The $x(\lambda)$ and $z(\lambda)$ curves determine the colour chromaticity. $x(\lambda)$

represents the mix of colour cones, while $z(\lambda)$ represents the blue colour cone. In the visible, i.e. in the 400-700 nm region, all the curves have non-zero values. For each spectrum the corresponding CIE XYZ values can be obtained with the following formulas:

$$X = \frac{100}{K} \int R(\lambda)\, x(\lambda)\, S(\lambda)\, d\lambda \tag{1}$$

$$Y = \frac{100}{K} \int R(\lambda)\, y(\lambda)\, S(\lambda)\, d\lambda \tag{2}$$

$$Z = \frac{100}{K} \int R(\lambda)\, z(\lambda)\, S(\lambda)\, d\lambda \tag{3}$$

The CIE XYZ values were linear transformed to RGB, in the form of fractional integers, ranging from 0-1, with

$$\begin{bmatrix} R \\ G \\ B \end{bmatrix} = \begin{bmatrix} 3.06332 & -1.39333 & -0.475802 \\ -0.969243 & 1.87597 & 0.0415551 \\ 0.678713 & 0.228834 & 1.06925 \end{bmatrix} \begin{bmatrix} X \\ Y \\ Z \end{bmatrix}$$

Subsequently, the obtained RGB values ranging from 0-1 are gamma corrected. The Gamma-correction applies a non-linear transform to the RGB values accounting for the non-linear response to luminance in human colour vision. The gamma-correction function is applied to the linear R, G, and B values, and is described by[3]

$$R_{sRGB} = \begin{cases} 12.92R & R \leq 0.0031308 \\ \left(1.055R^{\frac{1}{2.4}}\right) - 0.055 & R > 0.0031308 \end{cases}$$

$$G_{sRGB} = \begin{cases} 12.92G & G \leq 0.0031308 \\ \left(1.055G^{\frac{1}{2.4}}\right) - 0.055 & G > 0.0031308 \end{cases}$$

$$B_{sRGB} = \begin{cases} 12.92B & B \leq 0.0031308 \\ \left(1.055B^{\frac{1}{2.4}}\right) - 0.055 & B > 0.0031308 \end{cases}$$

The standard decoding gamma for sRGB is $\gamma = 2.2$. An exponent of 2.4 is used, to compensate for the linear threshold region below 0.0031308. This transform returns fractional (0-1) sRGB values, which are multiplied by 255 and rounded to generate a set of three 8-bit numbers ranging from 0-255. sRGB values combine luminance and chromaticity of a colour. In colour vision, the luminance indicates how much luminous power will be detected by an observer of an object or surface from a particular angle of observation. The larger the luminance value of the colour, the higher the visibility of that colour under the observation conditions. Chromaticity is an objective quantitative measure of the quality of colour, and is independent of the luminance value of the object or surface being observed. Chromaticity values represent all discernible different colours that can be perceived by humans using the three colour cones in the retina. High sRGB values indicate higher luminance values. For example,

a sRGB of 255:4:4 indicates a predominantly red colour of maximum luminance, and so would be visible as a bright, pure red colour to the human eye. A value of 63:1:1 also represents a red colour of the same chromaticity (as the relative weighting is the same) but at a quarter of the luminance, and so would be visible as a darker red colour.

For the CIE xyY plots, the XYZ primaries can also be mapped with the following functions[4]:

$$x = \frac{X}{X + Y + Z}$$

$$y = \frac{Y}{X + Y + Z}$$

The Y value in the xyY is the Y primary in the CIE standard observer functions, and denoted luminance.

3. Calculated colour generation (sRGB) for different polymer thicknesses

d (nm)	0	20	40	60	80	100	120	140	160	180	200	220	240	260	280	300	320	340	360	380
sRGB	248<br>111<br>56	240<br>111<br>94	211<br>110<br>144	178<br>129<br>178	143<br>171<br>187	139<br>194<br>149	195<br>166<br>98	254<br>135<br>42	216<br>99<br>4	179<br>80<br>46	190<br>56<br>110	198<br>48<br>159	189<br>93<br>167	172<br>143<br>139	173<br>163<br>97	197<br>144<br>64	230<br>127<br>57	255<br>100<br>90	228<br>62<br>131	191<br>59<br>154

Table S1: Calculated sRGB values of the generated colors for different polymer thicknesses.

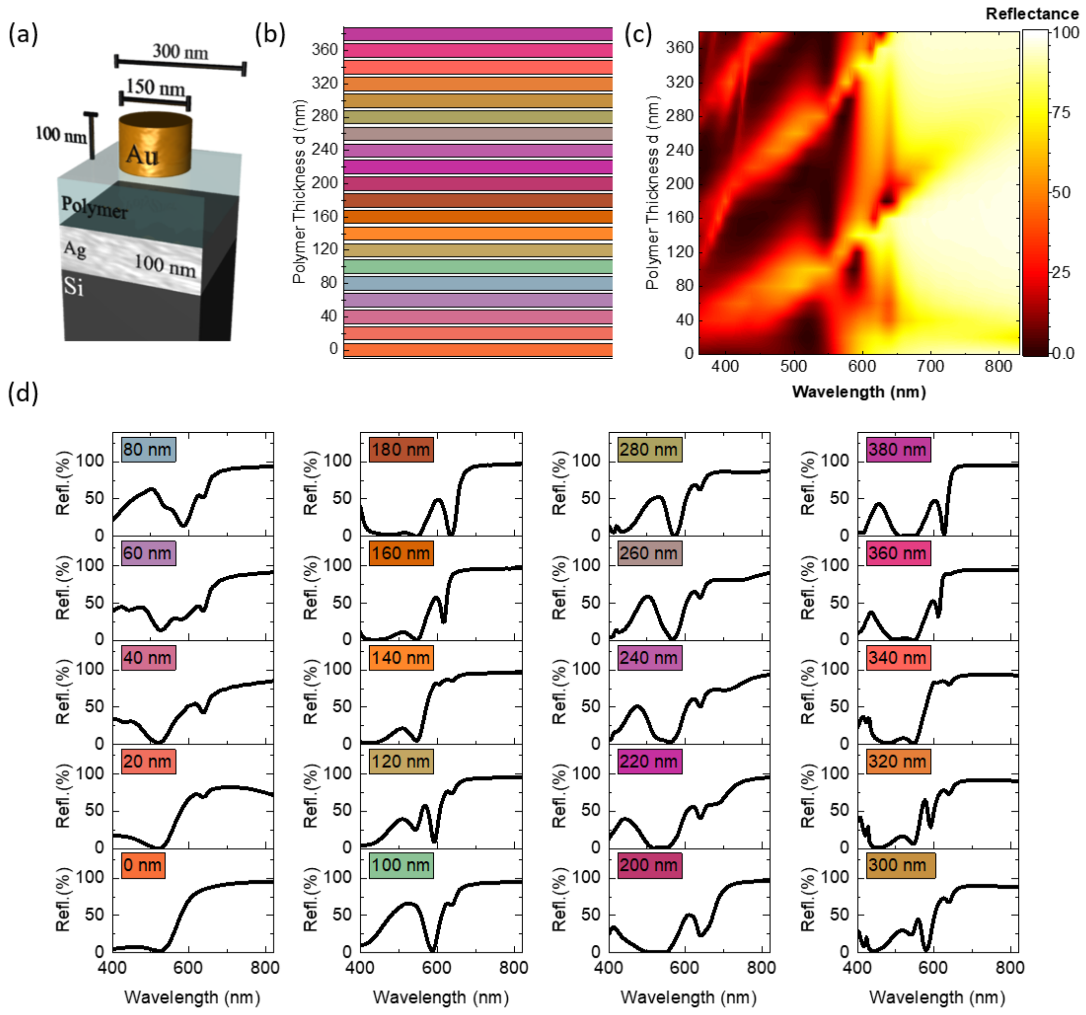

Figure S2: (**a**) Schematic of the Au nanodisc- Ag back-reflector hybrid nanostructure unit cell. The full structure is a square array (300 nm pitch) of gold nanodiscs (100 nm high and 150 nm in diameter) on a reflecting substrate consisting of a planar polymer thin film on top of a 100 nm thick silver (Ag) layer on silicon. (**b**) Calculated colour generation (sRGB) for different polymer thickness. (**c**) Simulated reflectance as a function of polymer thickness. (**d**) Simulated reflectance spectra of the hybrid structures for increasing polymer spacer thicknesses. The individual generated colours (sRGB) are displayed as insets.

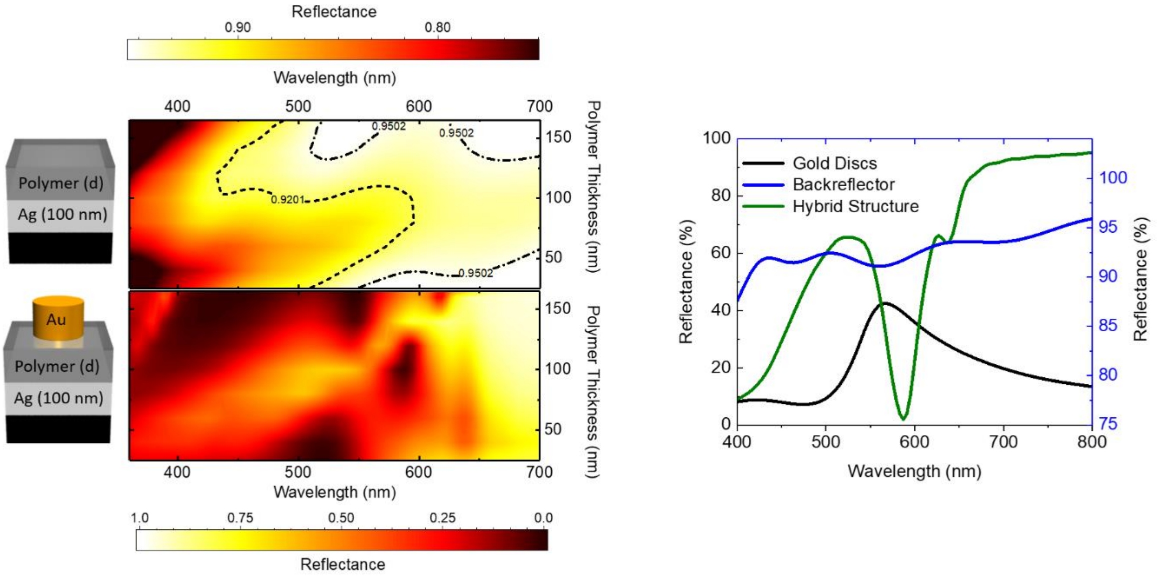

Figure S3: Comparison of the reflectance of the back-reflector alone with the reflectance from the Au nanodisc- Ag back-reflector hybrid structure and the simulated reflectance spectra of the individual components, a free standing array of gold discs (black spectra) and the backreflector substrate (blue spectra), as well as the reflectance spectra of the full hybrid structure combining the Au nanodisc array and the backreflector. The occurrence as well as absence of the spectrally narrow feature at 585 nm is governed by the interaction of localized plasmonic modes of the nanodiscs with the thin film modes, i.e. the spectral overlap with the minima in reflectance (dashed line) of the back-reflector.

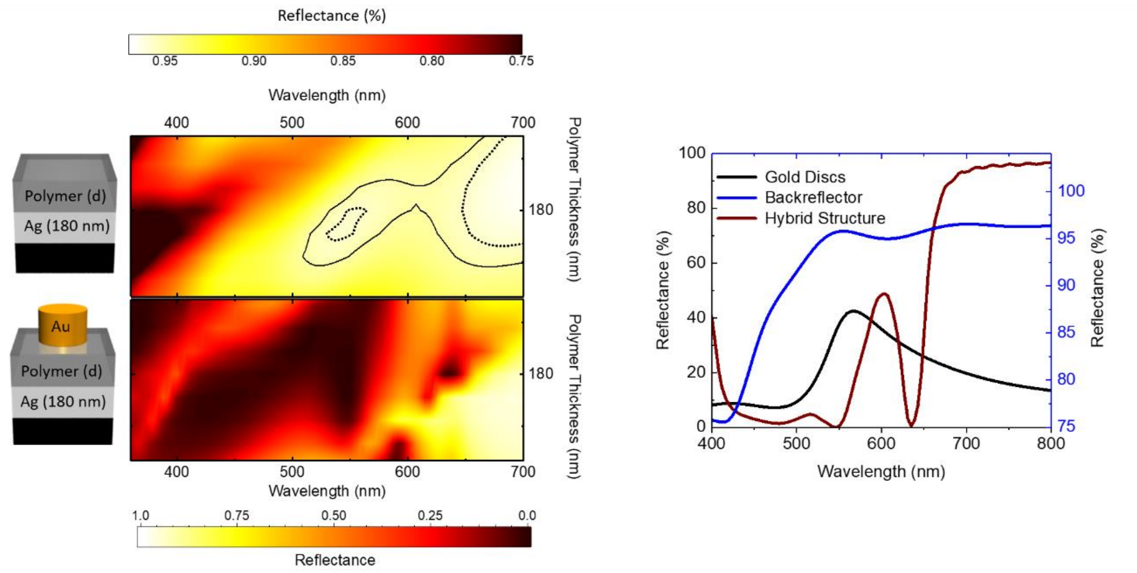

Figure S4: Comparison of the reflectance of the back-reflector alone with the reflectance from the Au nanodisc- Ag back-reflector hybrid structure and the simulated reflectance spectra of the individual components, a free standing array of gold discs (black spectra) and the backreflector substrate (blue

spectra), as well as the reflectance spectra of the full hybrid structure combining the Au nanodisc array and the backreflector. Also the occurrence as well as absence of the spectrally narrow feature at 625 nm is governed by the interaction of localized plasmonic modes of the nanodiscs with the thin film modes, i.e. the spectral overlap with the minima in reflectance (solid line 95.0 %) of the back-reflector. In contrast the absence of the spectrally narrow feature at 585 nm is governed by the interaction of localized plasmonic modes of the nanodiscs with the thin film modes, i.e. the spectral overlap with the maxima in reflectance (dashed doted line at 96.7%) of the back-reflector.

4. Main mechanisms, responsible for the colour generation

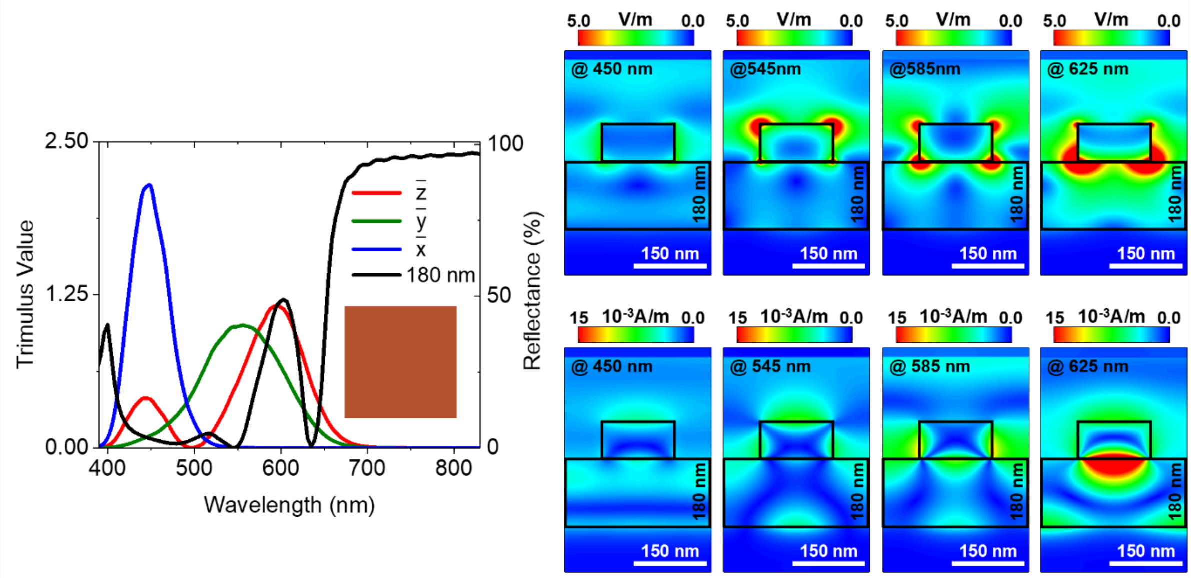

Figure S5: Main mechanisms for the generation of red and orange colours. The hybrid nanostructure with 180 nm polymer thickness was chosen as a typical example. Left: The calculated reflectance spectra of the hybrid nanostructure as well as the three equal area integral functions x(λ), y(λ) and z(λ), corresponding to the CIE XYZ 2° standard observer functions[1]. The generated colour is displayed as inset. Right: The electric and magnetic field distributions at 450 nm, 545 nm , 585 nm and 652nm. For a better visualisation, the field distributions are displayed on the same scale. Low reflectance in the spectral region between 400 and 550 nm is necessary for the generation of red and orange colours. In our nanostructures, the interaction between the plasmonic modes of the nanodisc arrays and the back reflector modes is the main mechanism responsible for the colour generation. At 450 nm and at 545 nm the light is trapped within the nanostructure, mostly at the nanodics air/ air polymer interface. Moreover, the electric fields exhibit multipolar character. Therefore, the reflectance is extremely low. As discussed in the main manuscript, the electric fields distributions around the nanoparticle exhibit dipolar character at 585 nm. The dipolar character of the localized nanodisc modes increases the reflectance in the red spectral region, and overlaps with the maximum of z(λ). At 625 nm, the light is completely trapped within the nanostructure, mostly in the polymer layer. However, the spectrally narrow feature at around 625 nm plays only a marginal role in the colour generation, due to the rather small spectral overlap with the redder tail of z(λ). The high reflectance above 700 nm does not spectrally overlap with the CIE XYZ 2° standard observer functions, and as expected is not crucial for the colour generation.

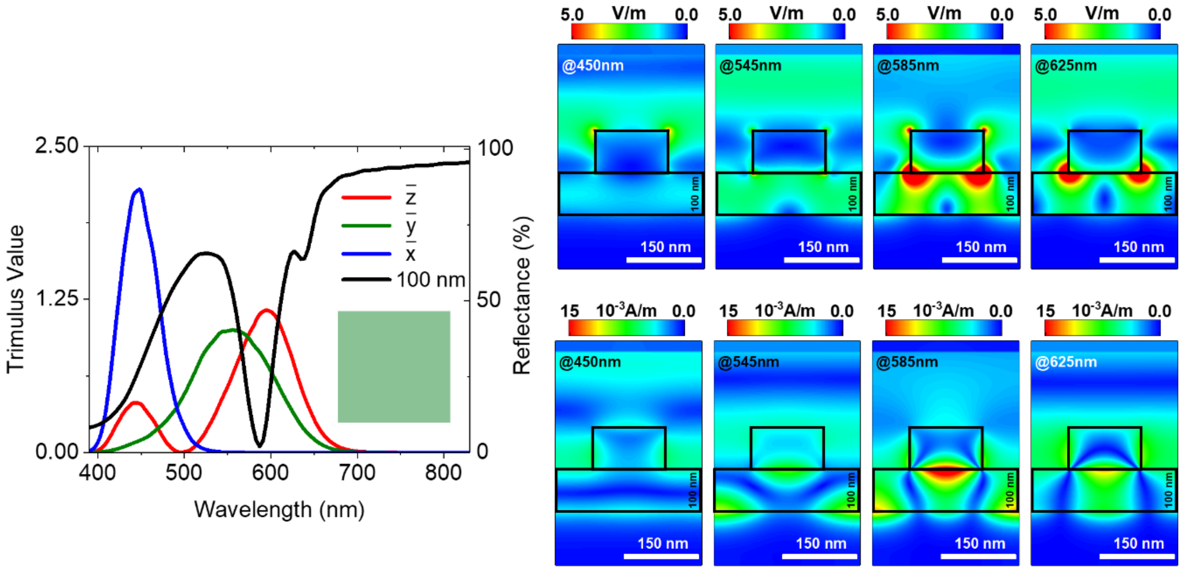

Figure S6: Main mechanisms for the generation of green colours. The hybrid nanostructure with 100 nm polymer thickness was chosen as a typical example. Left: The calculated reflectance spectra of the hybrid nanostructure as well as the three equal area integral functions x(λ), y(λ) and z(λ), corresponding to the CIE XYZ 2° standard observer functions[1]. The generated colour is displayed as inset. Right: The electric and magnetic field distributions at 450 nm, 545 nm , 585 nm and 652nm. For a better visualisation, the field distributions are displayed on the same scale. For the generation of green colours, a high reflectance between 500 and 550 nm is desirable. Additionally low reflectance in the spectral region between 400 and 500 nm and above 550 nm is necessary. At 450 nm and at 545 nm the back reflector modes are the main mechanism responsible for the relative high reflectivity. At 450 nm , the gold d-absorption reduces the reflectivity as well, which in this case is beneficial for the generation of green colours. The spectrally narrow feature at 585 nm overlaps with the maximum of z(λ). The extremely low reflectivity compensates the maximum of z(λ). Consequently, narrow feature at 585 nm is crucial for the generation of green colours. Green colour can be only generated with hybrid nanostructures with certain polymer thicknesses (100 nm and 280 nm), since this feature arises from both, the spectral and spatial overlap of the localized plasmonic modes of the nanodiscs and the local minima of the back reflector modes. In contrast, at 625 nm the reflectance seems dominated by the back-reflector mode, which at this wavelength exhibits a local maximum. Therefore, the reflectance is substantially higher at 625 nm, since the electric and magnetic fields couple now to the far field. Additionally, the electric and magnetic field amplitudes at 625 nm are smaller than at 585 nm, therefore the light trapping within the nanostructure is weaker, as discussed in the main manuscript.

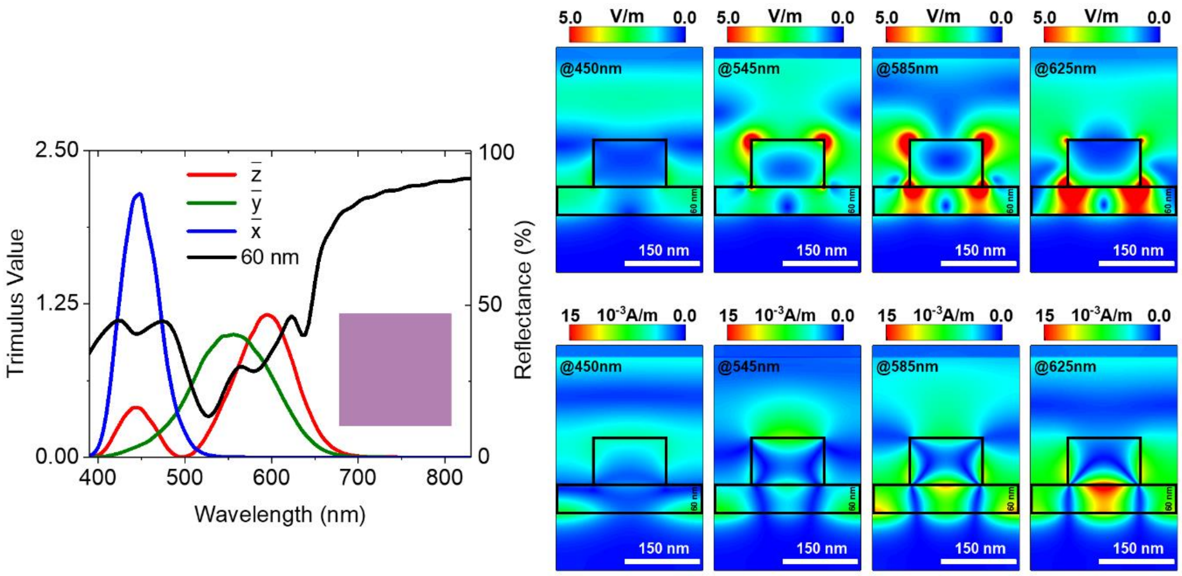

Figure S7: Main mechanisms for the generation of blue and violet colours. The hybrid nanostructure with 60 nm polymer thickness was chosen as a typical example. Left: The calculated reflectance spectra of the hybrid nanostructure as well as the three equal area integral functions x(λ), y(λ) and z(λ), corresponding to the CIE XYZ 2° standard observer functions[1]. The generated colour is displayed as inset. Right: The electric and magnetic field distributions at 450 nm, 545 nm , 585 nm and 652nm. For a better visualisation, the field distributions are displayed on the same scale. For the generation of blue colours, a high reflectance between 400 and 500 nm is desirable. Additionally low reflectance in the spectral region above 500 nm is necessary. At 450 nm the back reflector modes are the main mechanism responsible for the relative high reflectivity. At 450 nm, the gold d-absorption reduces the reflectivity as well, which in this case is detrimental for the generation of blue colours. Although, some of the light is trapped within the nanostructure at 545 nm, 585 nm and 625nm, for thin films 60-80 nm, the reflectance seems dominated by the back-reflector modes. The electric and magnetic fields couple now to the far field, increasing the reflectance. Additionally, the electric fields distributions around the nanoparticle exhibit dipolar character at 585 nm. Fortunately, the plasmonic coupling between the nanodiscs and the Ag film distorts the dipolar character, and hence it weakens the coupling to the far field. For substantially thicker polymers (220 nm or 260 nm) the light trapping is more efficient especially at 545 nm.

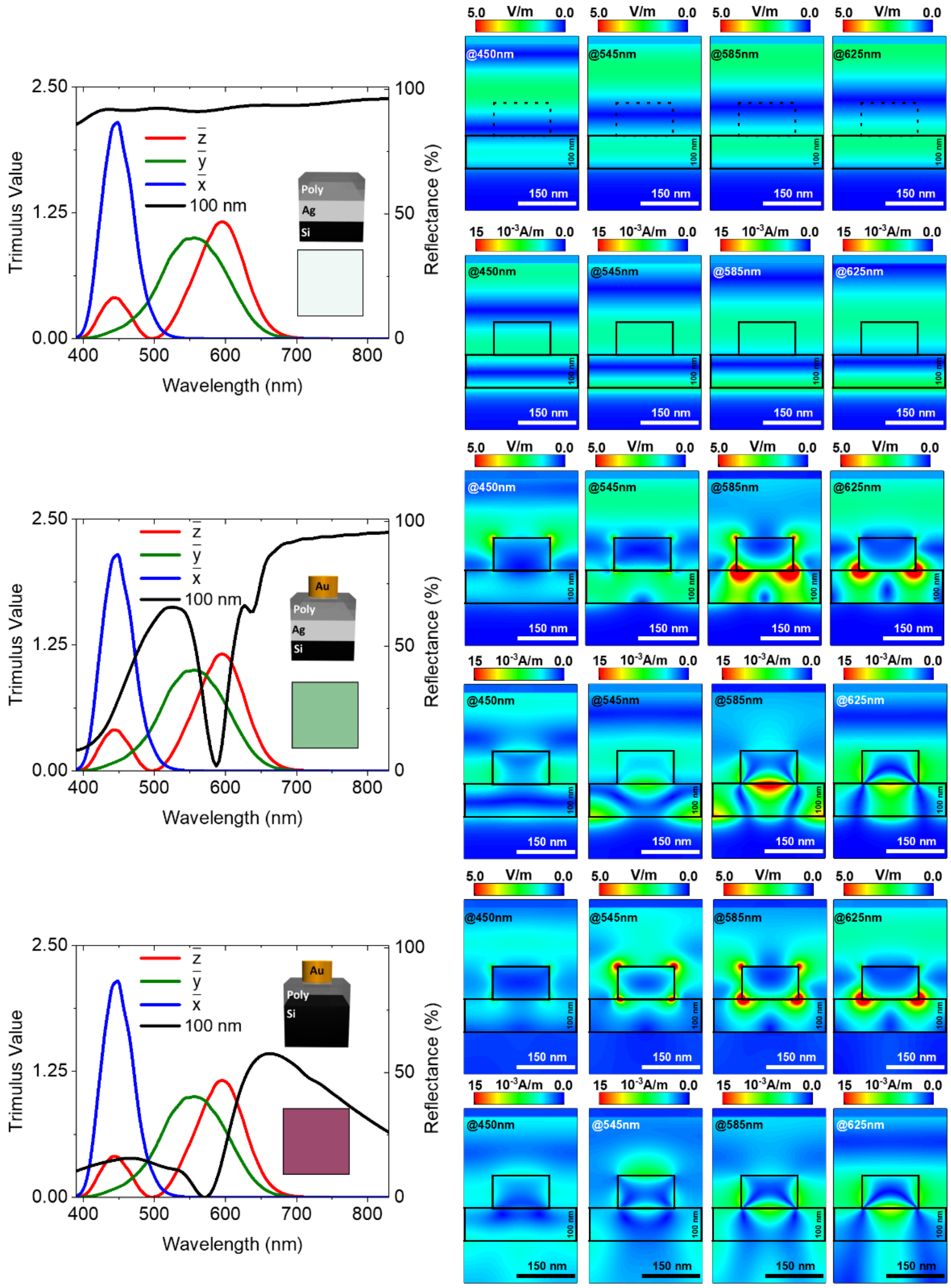

Figure S8: Left: The calculated reflectance spectra of the back reflector alone (Upper panel), of the hybrid nanostructure and of a hybrid nanostructure with the Ag thin film as a back reflector. The generated colour are displayed as inset. Right: The electric and magnetic field distributions at 450 nm, 545 nm, 585 nm and 625nm. The spectral narrow feature at 585 nm arises from both, the spectral and spatial overlap of the localized plasmonic modes of the nanodiscs and the local minima of the back reflector modes. The high reflectivity at 545 nm, crucial for green colour generation, vanishes if the Ag thin film is not present, since now the electric and magnetic fields at 545 nm are now confined around the nanodiscs.

5. Reflectance spectra for different angles of incidence

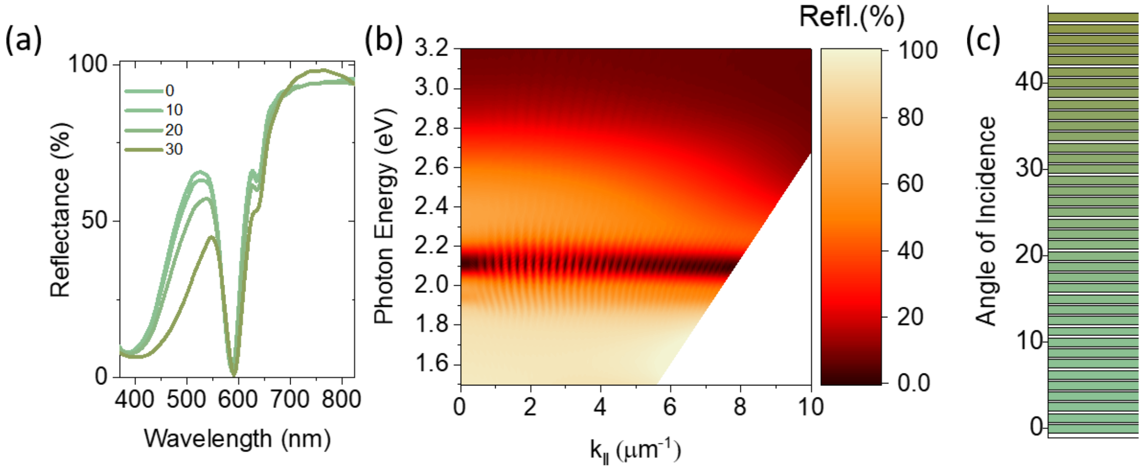

Figure S9: (**a**) Reflectance spectra for different angles of incidence. (**b**) Optical band structure of the Au nanodisc- Ag back-reflector hybrid structure with 100 nm polymer thickness, i.e. the calculated reflectance as a function of the in-plane wavevector and energy for p-polarized excitation. (**c**) Calculated colours (sRGB) over a range of angles of incidence.

6. Calculated colour generation (sRGB) for different nanodisc heights

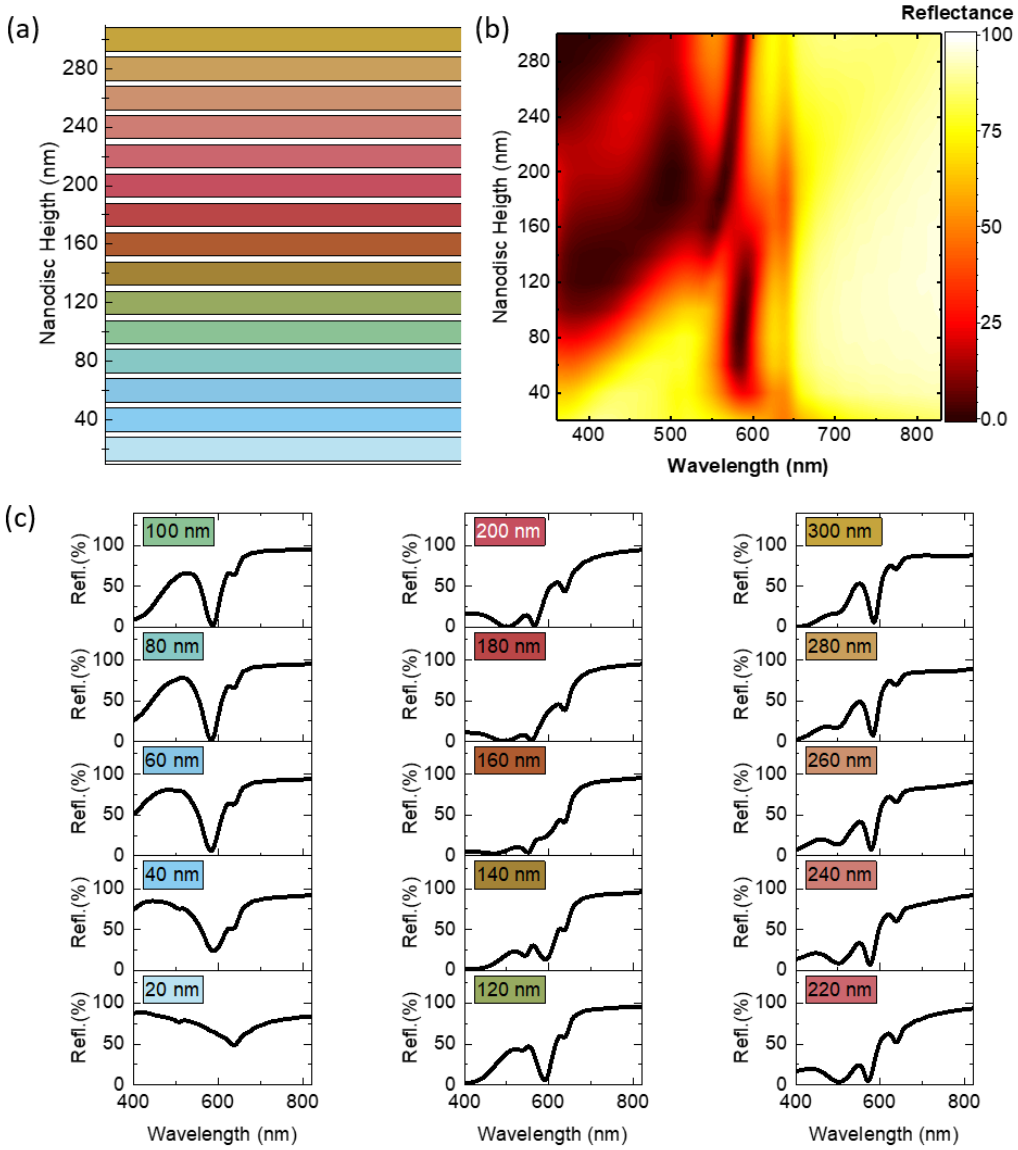

Figure S10 Au Nanodisc height dependence: (**a**) Calculated colour generation (sRGB) for different nanodisc heights. (**b**) and (c) Simulated reflectance of hybrid structures with increasing nanodisc height. (**c**) The sRGB colours corresponding to the individual spectra are displayed as an inset. For nanodisc heights between 40 nm and 120 nm pronounced minimum in reflection occurs at 585 nm. This minimum spectrally narrows (down to 35 nm) for nanodiscs heights between 80 nm and-100 nm and the reflectance drops down below 2%. Surprisingly, an increase of the disc height by 200 nm leads to similar spectral features as for nanodisc heights between 40 nm and 120 nm. However, the investigation of this apparent periodicity is beyond the scope of this paper.

7. Calculated reflectance spectra of nanodisc arrays for discs with 125 nm, 150 nm and 150 nm diameter

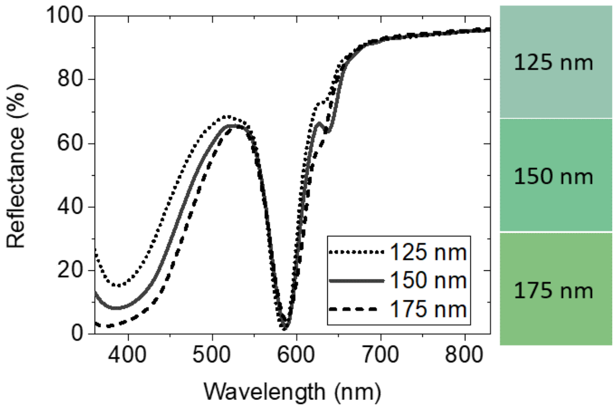

Figure S11 Au Nanodisc diameter dependence: Calculated reflectance spectra of nanodisc arrays for discs with 125 nm (dotted line), 150 nm (solid line) and 150 nm (dashed line) diameter. The spectral position and width as well as the magnitude of the main reflectance minimum are barely influenced by variation in the nanodisc diameter. The sRGB colours corresponding to the individual spectra are displayed right.

8. Comparison of the experimental and calculated colour generation.

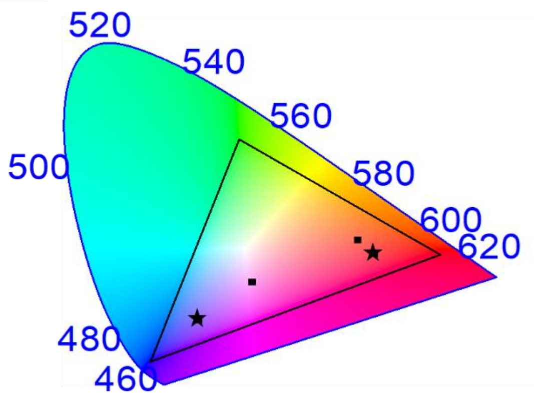

Figure S12 CIE Plot: .Comparison of the experimental (stars) and calculated colours (squares).

For the generation of blue colours, a high reflectance between 400 and 500 nm is desirable. Additionally low reflectance in the spectral region above 500 nm is necessary, as early discussed. The experimental spectra show these characteristics, while the calculated show higher reflectance and more defined features. The high reflectance in the green and red region in the calculated spectra results in a colour closer to the center of the CIE plot (Figure S10), while the experimental colour is closer to the blue primary. Although we attribute the differences mainly to variation in the thickness and surface roughness of the polymer layer, as well as to shape and size variation of the fabricated nanodiscs across the arrays. Additionally, the area underneath the discs could have been

overexposed due to the close spacing of the nanoparticles. As a consequence, the nanodiscs could be embedded in the polymer. The comparison of the simulated spectra for different embedding depth of the nanodiscs with the experimental data (Figure S11) suggest that this hypothesis is right. The calculated spectra for nanodiscs embedded 20 nm in the polymer film match the experimental spectra in the blue region, while the calculated spectra for nanodiscs embedded only 5 nm in the polymer are closer to the experimental values in the red spectral region. Consequently, one could conclude that the overexposure of the polymer area underneath the disc is not homogeneous and hence there is a gradient the polymer layer height underneath the nanodiscs.

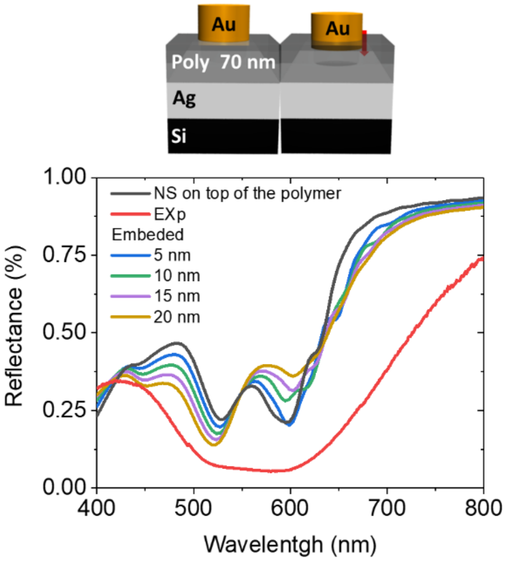

Figure S13 Calculated reflectance spectra of nanodisc arrays for discs with diameters of 175 nm and 100 nm in height. The nanodiscs could be embedded in the polymer to some extent, due to overexposure.

Low reflectance in the spectral region between 400 and 550 nm is necessary for the generation of red and orange colours. For the thicker sample, the well defined features above 625 nm observed in the calculated spectra play only marginal roles in the colour generation, due to the rather small spectral overlap with the redder tail of $z(\lambda)$. Especially the spectrally narrow feature at around 680 nm does not spectrally overlap substantially with the red CIE XYZ 2° standard observer functions and as expected is not crucial for the colour generation. Therefore, the calculated colours are close to the experimental observations.

Figure S12 (a) shows the dependence of the observed features at around 680 nm to variations of 5 nm in the thickness of the polymer layer as well as the spectra obtained from averaging the reflectance spectra for the three thicknesses. The nanodisc diameter is fixed to 175 nm. Figure S12 (b) shows the dependence of the observed features at around 680 nm to variations in the nanodiscs diameter and

the spectra obtained from averaging the reflectance spectra for the six diameters. The polymer layer thickness is fixed to 195 nm.

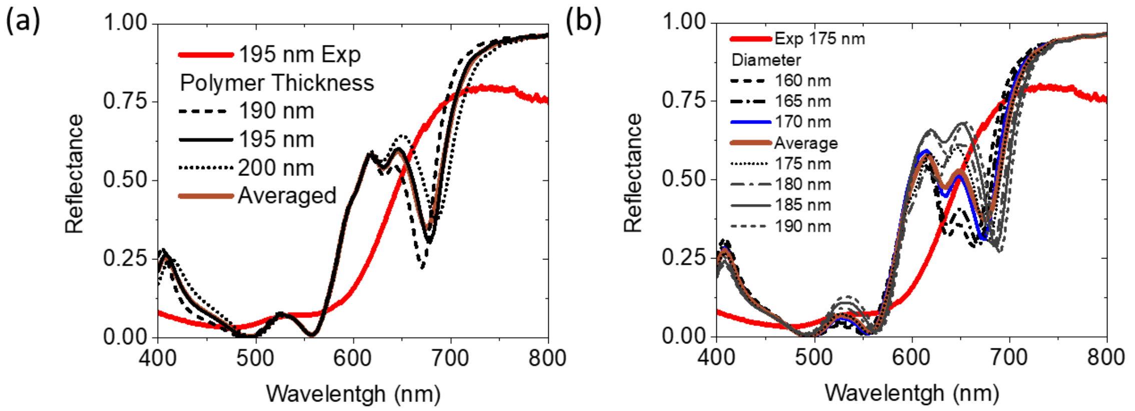

Figure S14 (**a**) Polymer thickness dependence and (**b**) Au Nanodisc diameter dependence: Calculated reflectance spectra of nanodisc arrays for discs with diameters ranging from 160 nm to 190 nm.

The spectrally narrow features above 625 nm are highly dependent on the disk diameter, in contrast to the spectrally narrow feature at 585 nm (**Figure S9**). As discussed previously, the spectrally narrow features arise from the interaction of localized plasmonic modes of the nanodiscs with the thin film modes, i.e., the spectral overlap with the minima in reflectance of the back-reflector. For thinner polymer films, i.e., 100 nm, the local minima and the local maxima in reflectance of the back-reflector are spectrally broader (cf. **Figure S3**) than the local minima and the local maxima observed for thicker polymer films, i.e, 180-200 nm (cf. **Figure S4**). Consequently, the spectral position of the spectrally narrow features above 625 nm observed for thicker films is strongly sensitive to changes in the spectral positions of the localized nanosdisc resonances, i.e., the red shifts of the resonances as the disc diameter is increased.

9. Calculated colour generation (sRGB) by a Cu nanodisc – Al back-reflector hybrid nanostructure as a function of polymer thickness.

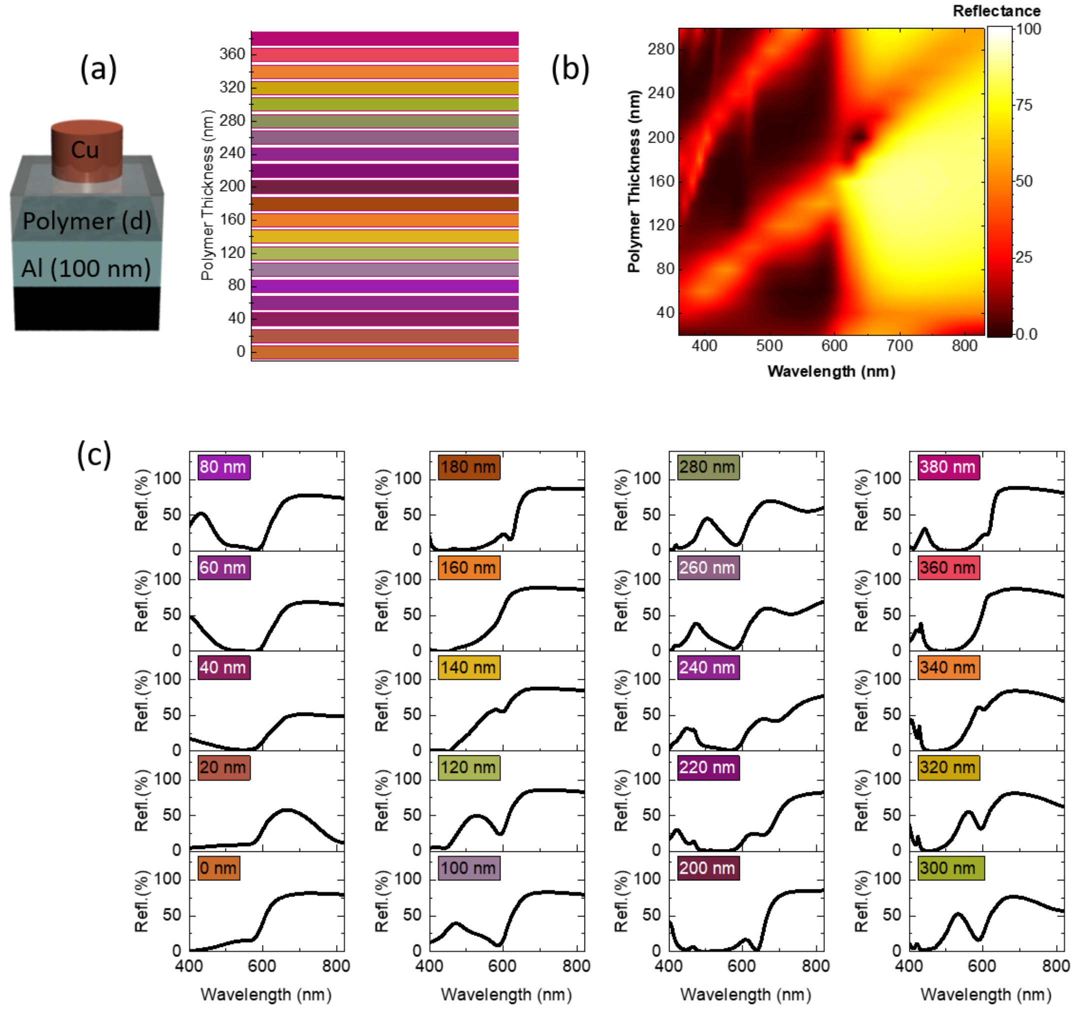

Figure S15: (**a**) Schematic of the Cu nanodisc – Al back-reflector hybrid nanostructure unit cell. The full structure is a square array (300 nm pitch) of copper nanodiscs (100 nm high and 150 nm in diameter) on a back-reflector substrate consisting of a polymer thin film on top of a 100 nm thick aluminium (Al) layer on silicon. Calculated colour generation (sRGB) for different polymer thickness. (**b**) Simulated reflectance as function of polymer thickness. (**c**) Simulated reflectance spectra of the hybrid structures for increasing polymer spacer thicknesses. The individual generated colours (sRGB) are displayed as insets.

10. References

1. International Commission on Illumination *Colorimetry : understanding the CIE system*; Schanda, J., Ed.; CIE/Commission internationale de l'eclairage, 2007; ISBN 9780470175620.

2. Choudhury, R.; Kumar, A. Principles of colour and appearance measurements; Woodhead, 2014;

3. Oleari, C. *Standard colorimetry*; Oleari, C., Ed.; John Wiley & Sons, Ltd: Chichester, UK, 2015; ISBN 9781118894477.

4. Kerr Issue, D.A. The CIE XYZ and xyY Color Spaces; 2010;
